# FLUORESCENCE LIFETIME PATTERNS IN MACULAR TELANGIECTASIA TYPE 2

**DOI:** 10.1097/IAE.0000000000002411

**Published:** 2019-01-03

**Authors:** Yasmin Solberg, Chantal Dysli, Sebastian Wolf, Martin S. Zinkernagel

**Affiliations:** * Department of Ophthalmology, Inselspital, Bern University Hospital, Bern, Switzerland; and; † Department of BioMedical Research, University of Bern, Bern, Switzerland.

**Keywords:** fluorescence lifetimes, fundus autofluorescence, ophthalmic imaging, MacTel, macular telangiectasia type 2, macula

## Abstract

Type 2 idiopathic macular telangiectasia (MacTel) is a rare bilateral neurodegenerative disease characterized by alterations in the macular capillary network leading to central vision loss. The purpose of this study was to quantify disease-specific retinal fluorescence lifetime patterns in patients with MacTel using fluorescence lifetime imaging ophthalmoscopy.

**Participants:**

Both eyes of 14 patients (mean age ± SEM, 67.8 ± 6.4 years) with a clinical diagnosis of MacTel Type 2 and 14 healthy age-matched controls (age 69.8 ± 6.4 years) were included in this study.

**Methods:**

All participants were imaged with a fluorescence lifetime imaging ophthalmoscope (Heidelberg Engineering, Germany). Mean retinal fluorescence lifetimes (Ƭm) were obtained in the short spectral channels (498–560 nm) and long spectral channels (560–720 nm). Clinical features, fundus images, fundus autofluorescence intensity images, spectral domain optical coherence tomography, and corresponding macular pigment optical density measurements using a modified confocal scanning laser ophthalmoscope (mpHRA) were further analyzed. Patients were classified into five phenotypic subgroups using the Gass and Blodi classification.

**Results:**

Mean fluorescence lifetimes were significantly prolonged temporal to the fovea in patients with MacTel compared with healthy controls (mean ± SEM: short spectral channels 543 ± 61 ps vs. 304 ± 9 ps; *P* < 0.0001; long spectral channels: 447 ± 26 ps vs. 348 ± 11 ps; *P* < 0.0001), and appeared as a crescent or ring-shaped pattern. Prolonged lifetime patterns correlated with decreased macular pigment density on macular pigment optical density measurements. Follow-up examinations were performed in four MacTel patients, which revealed an increase of short spectral channel Ƭ_m_ of 22% over 2.1 years in the temporal fovea.

**Conclusion:**

This study confirms that fundus autofluorescence lifetimes display characteristic patterns in patients with MacTel Type 2 disease and provide information about macular pigment and possibly photoreceptor loss. Fluorescence lifetime prolongation correlates with disease severity and may therefore be a useful addition to other imaging modalities for assessing disease progression in MacTel Type 2.

## Abstract 2

Supplemental Digital Content is Available in the Text.

This study confirms that fundus autofluorescence lifetimes display characteristic patterns in patients with MacTel Type 2 disease and provide information about macular pigment reduction and possibly photoreceptor loss. Fluorescence lifetime imaging ophthalmoscopy offers a novel diagnostic tool in the investigation of eyes with MacTel and has the potential of becoming a noninvasive screening modality.

Idiopathic macular telangiectasia Type 2 (MacTel) is a rare bilateral neurodegenerative disease, characterized by alterations of the retinal capillary network in the juxtafoveal region that usually manifests with a slowly progressive decrease in visual acuity with an onset in the fifth to sixth decades of life.^1–3^

The prevalence of the disease has been estimated to be as high as 0.1% among subjects aged 40 years and older.^4^ Despite recent advances in the understanding of the pathogenesis of this disease such as the identification of genetic loci associated with MacTel,^5^ many aspects of the disease still remain unknown. Histopathological studies have established that Müller cell degeneration^6^ in the central retina occurs in the same pattern as macular pigment depletion. This suggests that neuroglial loss, resulting in impairment cone and rod photoreceptor function, plays a central role in the pathogenic mechanism of the disease.^7,8^ Clinically, early manifestations of the disease include blunting of the foveolar reflex, loss of retinal transparency, crystalline deposits in the superficial retinal layers, and the presence of intraretinal cavities at the fovea. Retinal alterations and abnormal macular pigment levels initially manifest temporal to the fovea, whereas at later stages, an oval area centered approximately 6° horizontally and 5° vertically on the fovea is affected.^3,9^ At advanced stages, lack of macular pigment,^10^ formation of pigmented plaques along with vascular remodeling, fibrosis, and eventually subretinal neovascularization^2^ may occur and may limit the prognosis of central vision. To date, no effective treatment to modify the course of disease in nonproliferative MacTel Type 2 is available.

Multiple imaging modalities have been established as important parameters for assessing disease progression in MacTel, including fluorescein angiography, color fundus imaging, fundus autofluorescence (FAF) intensity imaging, optical coherence tomography (OCT), and confocal blue reflectance imaging.^11–13^

Fluorescence lifetime imaging ophthalmoscopy (FLIO) is an imaging technology supplementing the current armamentarium of multimodal retinal imaging.^14–17^ Because the diagnosis of MacTel can be challenging especially at early stages of disease, FLIO may serve as an additional tool for early detection of MacTel and possibly obtain information on disease progression. Because FLIO has been shown to detect changes in macular pigment with high contrast^18–20^ changes in macular pigment specific lifetimes provide information about macular photoreceptor or possibly Müller cell loss. In a recent study, MacTel-specific fluorescence lifetime patterns have been reported.^20^ The purpose of this study was to confirm these findings, to quantify disease-specific lifetime patterns in patients with MacTel, and to provide longitudinal fluorescence lifetime data.

## Methods

This prospective, cross-sectional study was conducted at the University Hospital in Bern, Switzerland, with the approval of the local ethics committee. The study was performed in accordance with International Conference on Harmonisation-Good Clinical Practice (ICH-GCP) guidelines, which correspond to the Health Insurance Portability and Accountability Act of 1996 (HIPAA) regulations, which is in accordance with the Declaration of Helsinki. This study is registered at ClinicalTrials.gov (NCT01981148). All patients and healthy controls were recruited at the Ophthalmology outpatient department of the University Hospital, Bern, Switzerland. An informed consent was signed before study entry.

Eyes of participants with a confirmed diagnosis of either nonproliferative or proliferative MacTel Type 2 were included in this study. Eyes with other retinal pathologies affecting the macula, such as choroidal neovascularization secondary to age-related macular degeneration, diabetic retinopathy, and retinal vein occlusion were excluded from the study.

All patients and healthy subjects underwent a comprehensive ophthalmologic examination with a measurement of the best-corrected visual acuity (BCVA; Early Treatment Diabetic Retinopathy Study [ETDRS] letters and fundus examination). Before imaging, maximal pupil dilation was achieved using tropicamide 0.5% and phenylephrine HCl 2.5%. Of both eyes, color fundus images (Zeiss FF 450plus; Zeiss, Oberkochen, Germany), fluorescein angiography, FAF images, and OCT scans of the macula (Heidelberg Spectralis HRA + OCT; Heidelberg Engineering, Heidelberg, Germany), macular pigment optical density (MPOD) measurements using a modified confocal scanning laser ophthalmoscope (mpHRA; Heidelberg Engineering), and fluorescence lifetime images (FLIO) were obtained. The Gass and Blodi classification was used categorize patients into phenotypic subgroups. Briefly, Stage 1 presents with occult vascular changes, Stage 2 with no clinically visible telangiectasia, Stage 3 with prominent and dilated retinal venules, Stage 4 with retinal pigment clumping, and Stage 5 with subretinal neovascularization.^9^

### Fluorescence Lifetime Imaging Ophthalmoscope

A fluorescence lifetime imaging ophthalmoscope based on existing Heidelberg Engineering Spectralis technology was used to acquire retinal fluorescence lifetime images from a 30° retinal field. The principles and safety details of FLIO have been described previously.^18^

In summary, the technology is based on the excitation of retinal FAF using a pulsed diode laser (473 nm, 80 MHz). Two highly sensitive hybrid photon-counting detectors (HPM-100-40; Becker & Hickl, Berlin, Germany) were used for detecting and measuring fluorescence photons in two separate wavelength spectrums: a short spectral channel (SSC: 498–560 nm) and a long spectral channel (LSC: 560–720 nm). Time-correlated single-photon counting modules (SPC-150; Becker & Hickl) were used. In both wavelength channels, at least 1,000 photons per pixel were obtained to ensure reliable image quality. A high-contrast confocal infrared image eye movement tracking system ensured that the correct location of each photon was detected. The chi-square value evaluated the exponential fit.

The mean fluorescence lifetime Ƭm was calculated using the short and long lifetime components (Ƭ1 and Ƭ2, respectively) with their relative amplitudes α1 and α2.

### Fluorescence Lifetime Data Analysis

The FLIO reader software (ARTORG Center for Biomedical Engineering Research, University of Bern, Switzerland) was used for analyses of all mean fluorescence lifetime data. For each eye, an FAF intensity image with its correlating pseudocolor fluorescence lifetime image was performed. For quantification of retinal autofluorescence lifetimes, a standard ETDRS grid was used with a circle diameter of 1 mm for the central area/fovea, 3 mm for the inner ring, and 6 mm for the outer ring.

### Statistical Analysis

The mean fluorescence lifetime data were analyzed for both spectral channels. Data were presented as mean ± SEM. To compare results and test for significant differences between groups, a Mann–Whitney nonparametric test was used. Statistical analysis was performed using GraphPad (Prism 6; GraphPad Software Inc, La Jolla, CA).

## Results

Fourteen consecutive patients (28 eyes) with a clinical diagnosis of MacTel Type 2 (mean age: 67.8 ± 6.4 years, 64.3% male) and 28 eyes of 14 healthy subjects (mean age 69.8 ± 6.4 years) were included in this study (60% male). All participants were white and had no concomitant ophthalmic diseases. One eye was pseudophakic. The mean visual acuity was 67 ± 3 ETDRS letters. Concerning systemic diseases, in patients with MacTel Type 2, the prevalence of arterial hypertension was 53.3%, hypercholesterolemia 50%, and diabetes mellitus 35.7%. Four patients (28.6%) reported to be ex-smokers. Follow-up examinations were performed in four (28.6%) MacTel patients. Further details about the investigated groups can be found in Table 1.

**Table 1.**
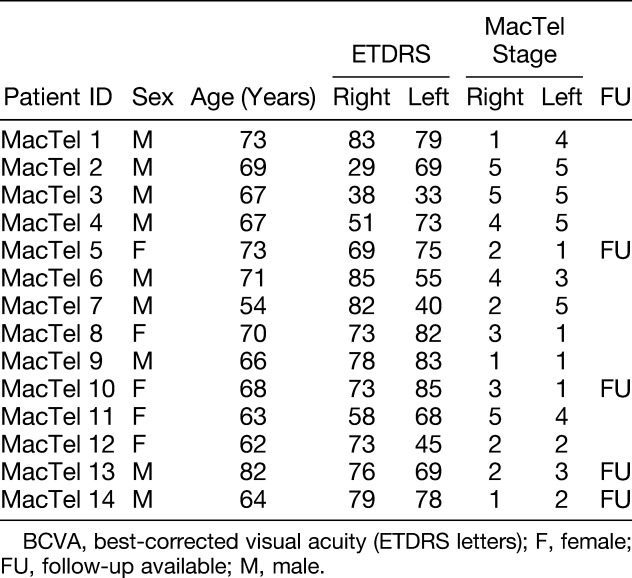
Patient Characteristics

### Autofluorescence Lifetimes in Patients With MacTel Type 2

**Fig. 1.**
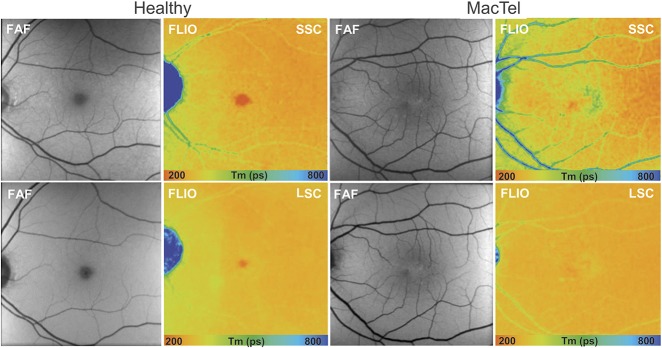
Fundus autofluorescence lifetime (color) and intensity (gray) images from the SSC (498–560 nm) and the LSC (560–720 nm) in a healthy subject and a patient with MacTel showing prolongation (blue) of mean fluorescence lifetime temporal to the fovea, with a reduction of short lifetimes (red) within the fovea.

The characteristic epicenter of the disease, located temporal to the fovea, colocalized with distinct autofluorescence lifetimes in FLIO. In the temporal parafoveolar area, we identified prolonged mean retinal autofluorescence lifetimes in a wedge-shaped pattern of long fluorescence lifetimes at early stages of MacTel, whereas at advanced stages, an oval ring-shaped lesion around the fovea was observed. Generally, the fluorescence lifetime contrast of disease-specific changes was best seen in the SSC (Figure 1).

When comparing autofluorescence lifetime differences between patients with MacTel Type 2 and healthy participants, there was a general prolongation of mean fluorescence lifetimes.

**Fig. 2.**
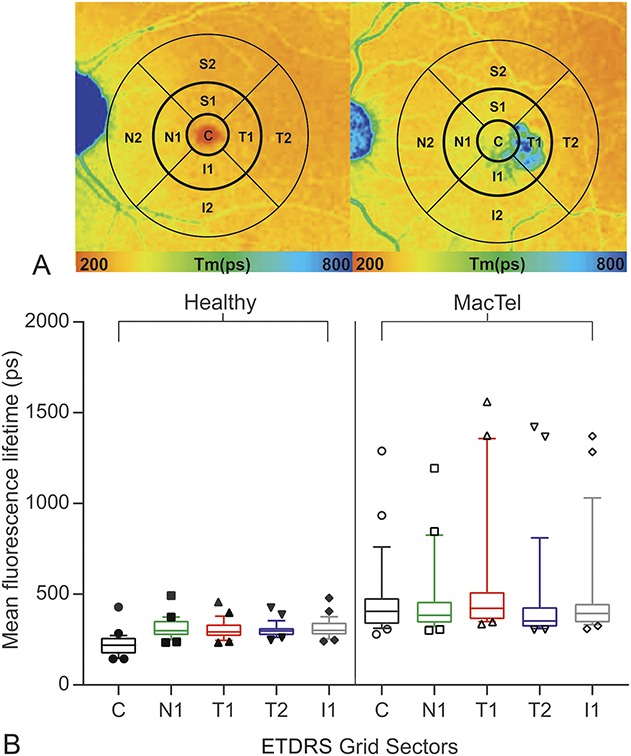
**A.** Control eye from healthy subject and MacTel eye with indicated way of data analysis. A standard ETDRS grid (center [diameter; d = 1 mm], inner [d = 3 mm], and the outer [d = 6 mm] ETDRS ring) was used for data averaging. FLIO SSC = 498 mm to 560 nm, fluorescence lifetime images of the SSC. **B.** Quantitative analysis of mean retinal autofluorescence lifetime values in the SSC in healthy subjects and MacTel patients. Box plots of mean fluorescence lifetimes (Ƭm) with 10 to 90 percentile of the following areas of the ETDRS grid: central (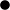), nasal sector (N1, 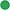), temporal sector (T1, 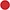), temporal outer sector (T2, 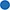), inferior sector (I1, 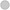).

In particular, within the MacTel area (inferotemporal region), significant differences were found (*P* < 0.0001) compared with the corresponding area in the healthy control eyes (Figure 2).

In the MacTel cohort, mean lifetime values Ƭ_m_ within the inner ring of the ETDRS grid were 488 ± 47 ps in the SSC and 410 ± 13 ps in the LSC. Thereby, Ƭ_m_ was lengthened by 55% in the SSC and by 31% in the LSC compared with healthy control eyes (*P* < 0.001 and *P* < 0.01, respectively).

In MacTel eyes, mean lifetimes of T1 (SSC 543 ± 61 ps) were prolonged compared with T2 (301 ± 7 ps) in healthy controls, suggesting that additional factors to macular pigment loss such as degeneration of photoreceptors or lipofuscin accumulation lead to longer mean lifetimes in T1 of MacTel patients.

The longest autofluorescence lifetimes were identified in eyes affected by MacTel within the temporal quadrant (T1), followed by the inferior (I1), superior (S1), and the nasal quadrants (N1) (Figure 2). Compared with the healthy control population, the fluorescence lifetimes were significantly prolonged in the temporal quadrant in both spectral channels (SSC 543 ± 61 ps vs. 304 ± 9 ps, *P* < 0.0001; LSC: 447 ± 26 ps vs. 348 ± 11 ps, *P* < 0.0001).

Within the MacTel population, there was a significant difference (*P* = 0.0093) in retinal fluorescence lifetime between the temporal paracentral region compared with the nasal sector. By contrast, a reversed pattern was identified in healthy subjects, with the temporal sector displaying shorter lifetimes in comparison with the nasal sector (SSC: 304 ± 10 ps, LSC: 348 ± 11 ps vs. SSC: 322 ± 11 ps, LSC: 348 ± 11 ps).

### Fluorescence Lifetimes at Different Severity Grades

We analyzed differences in autofluorescence lifetimes between healthy participants and patients with MacTel at five different severity grades according to the Gass and Blodi classification.^1,9^ Disease severity was graded Stage 1 in 25% (7) eyes, Stage 2 in 21% (6) eyes, Stage 3 in 14% (4) eyes, Stage 4 in 14% (4) eyes, and Stage 5 in 25% (7) eyes.

The ETDRS subfields were correlated with disease severity (Table 2). Analysis of data in early-stage MacTel (Stage 1) revealed that in SSC, the mean fluorescence lifetimes was significantly prolonged in ETDRS sectors T1 (*P* = 0.04), C (*P* < 0.0001) and I1 (*P* = 0.0046), in comparison to the healthy control group, whereas differences in sectors N1 (*P* = 0.42) or T2 (*P* = 0.3) were not significant. In the LSC, we found no significant change in any ETDRS sectors between healthy controls and Stage 1 MacTel patients.

**Table 2.**
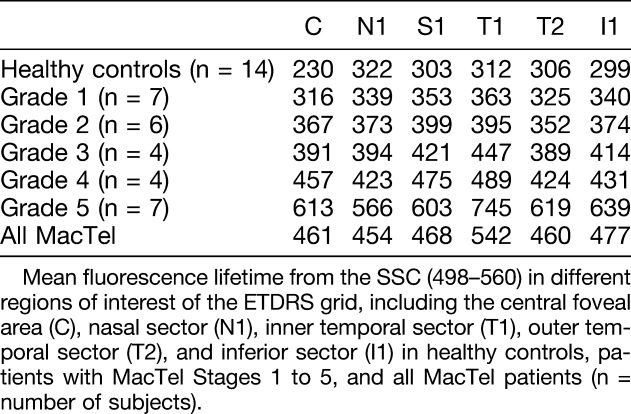
Fundus Autofluorescence Lifetimes in MacTel Patients and Healthy Subjects

The mean fluorescence lifetime in the SSC measured in areas of T1 were compared with N1 values. In healthy controls, the mean autofluorescence lifetime of the T1 sector was 3.2% shorter when compared with the N1 sector. However, in eyes with MacTel, T1 was prolonged in Stage 1 by 6.9%, in Stage 2 by 5.4%, in Stage 3 by 11.5%, in Stage 4 by 15.7%, and in Stage 5 by 21.3% when compared with mean lifetimes in N1, respectively.

### Fluorescence Lifetime Imaging Ophthalmoscopy and Multimodal Imaging Features

All patients displayed characteristic disease features of MacTel Type 2 in FAF, OCT, and fundus photography.

In OCT, MacTel-specific findings as described below presented in various combinations but were most prominent temporal to the fovea.

**Fig. 3.**
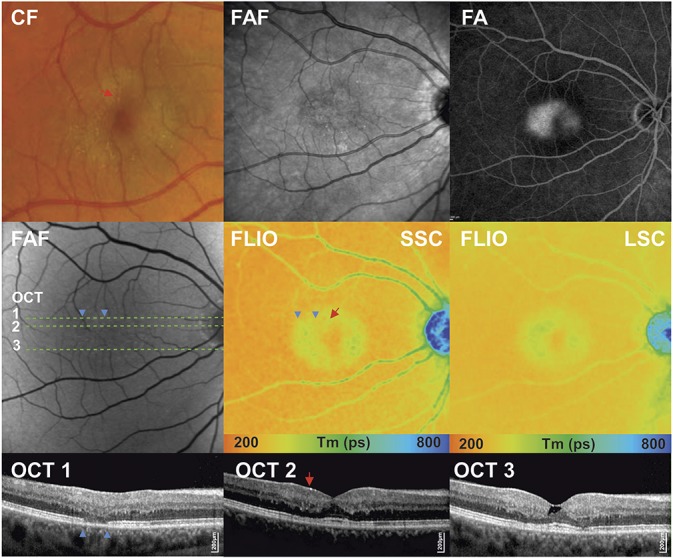
Multimodal imaging of a patient with MacTel Type 2 in a nonproliferative stage. Fundus photography (color fundus [CF]) showing parafoveal loss of retinal transparency and retinal crystals; fundus autofluorescence image (FAF) showing mild increase of foveal autofluorescence; fluorescence angiogram (FA) with late-phase leakage; spectral domain OCT horizontal scans 1 to 3, with indicated green line.

The most common OCT finding in this study was parafoveal hyporeflective spaces. These spaces corresponded to areas of prolonged lifetimes located within the perifoveal oval- or wedge-shaped region on topographic FLIO maps. In addition, rarefaction of the ellipsoid zone was a frequent finding. Areas with ellipsoid zone loss generally featured longer Ƭ_m_ (Figure 3). Four eyes had evidence of crystalline deposits on fundus photography and clinical examination. Crystals were identified on fundus images and were colocalized within the nerve fiber layer on OCT. The areas with crystalline deposits were then investigated with FLIO. Crystalline deposits featured distinctively prolonged mean lifetimes, which were most apparent in the short-wavelength channel, but still discernible in the long-wavelength channel (Figure 3).

In advanced stages of MacTel (Stages 4), sharply demarcated, parafoveal hypofluorescent lesions seen on FAF, corresponding to hyperpigmented areas on fundus photography, were observed and correlated with areas of prolonged autofluorescence lifetimes in FLIO. Neovascular membranes (Stage 5) seen as a thickening of the hyperreflective layer on OCT also corresponded with circular lesions, which led to pronounced prolongation of fluorescence lifetimes.

**Fig. 4.**
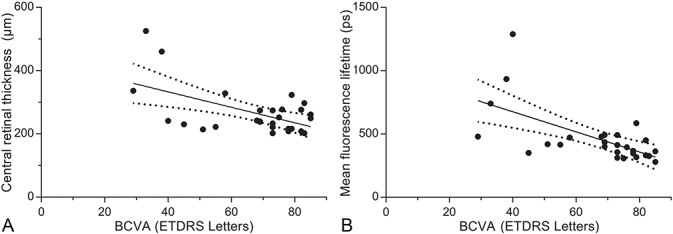
**A.** Correlation of BCVA (ETDRS letters) with central retinal thickness (r^2^ = 0.301, *P* = 0.0025). **B.** Correlation of BCVA (ETDRS letters) with mean fluorescence lifetimes of the SSC (r^2^ = 0.39, *P* = 0.0004).

The mean retinal thickness of the central subfield was 266 ± 14 µm and showed a correlation with the BCVA, whereby increased retinal thickness was associated with poorer ETDRS letter scores (r^2^ = 0.301, *P* = 0.0025). Mean fluorescence lifetimes of the central subfield of the SSC also showed correlation with BCVA, whereby increased lifetimes were associated with a poorer ETDRS letter score (r^2^ = 0.39, *P* = 0.0004; Figure 4).

### Autofluorescence and Macular Pigment

In healthy subjects, the shortest fluorescence lifetimes were seen within the macula, visualized as a red color-coded signal on FLIO, probably originating from macular pigment. When we compared mean fluorescence lifetimes from the central subfield of the ETDRS grid of all MacTel patients with those of healthy age-matched controls, Ƭm was prolonged by 107% in the SSC (*P* = 0.0001) and by 30% in the LSC (*P* = 0.0016). However, patients with MacTel Stage 1 had less changes in Ƭm of 48% in the SSC (*P* = 0.0417) and 30% in the LSC (*P* = 0.1356).

Macular pigment optical density measurements were obtained of 21 eyes with MacTel Type 2. In general, patients displayed an abnormal distribution with a reduced central MPOD signal and a surrounding ring of increased density at about 6° eccentricity.

**Fig. 5.**
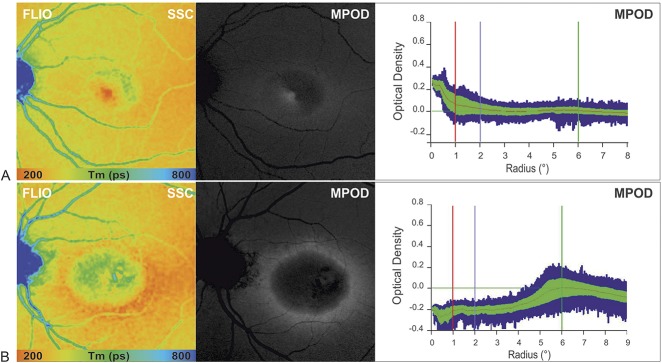
Comparison of MPOD profiles obtained using 2-wavelength FAF in two patients with MacTel Type 2 showing reduced central macular pigment with corresponding FAF lifetime images in the SSC (FLIO, SSC = 498–560 nm) (**A**) at MacTel Stage 2 residual macular pigment (MP) at the fovea, and (**B**) at MacTel Stage 5 with a surrounding ring of preserved MPOD at about 6° eccentricity.

In general, in this study, MacTel patients exhibited a reduced central macular pigment signal in comparison with healthy controls as measured by a confocal scanning laser ophthalmoscope (mpHRA). Although in some patients a central peak in MPOD measurements could still be found, the short fluorescence lifetime signal appeared irregularly shaped and smaller in comparison with healthy eyes. At more advanced stages, recordings displayed short fluorescence lifetimes in a ring-like dispersion pattern surrounding the parafoveal area. Prolonged lifetime patterns correlated with decreased macular pigment density on MPOD measurements (Figure 5).

### Analysis of Individual Fluorescence Lifetime Components

We investigated the influence of individual lifetime components (Ƭ1 and Ƭ2) and their corresponding amplitudes (α1 and α2) to observe changes seen in Ƭ_m_.

Hyperpigmented plaques seen on fundus photography could be isolated from the surrounding retina, due to higher Ƭ1 and Ƭ2 components in comparison with the surrounding retina. Our data revealed that short fluorescence lifetimes (color coded red) could be isolated at different stages of the disease. At early stages, short lifetimes were found in the foveal center. As the disease progressed, short lifetimes were less evident in the macular region but appeared in a ring-like structure surrounding the fovea. Shorter lifetimes had a higher Ƭ1 component than the surrounding retina.

The MacTel area can be visualized using phasor plots (see **Figure 1, Supplemental Digital Content 1**, http://links.lww.com/IAE/A944).^21^ The phasor plot demonstrates the association between the short and the long decay components, where short variables are located to the right side of the semicircle and long components toward the left side. The mean autofluorescence lifetime values measured are dispersed on a virtual line between the short and the long components. In the phasor plot, we observed a lifetime cloud correlating with the foveal area in healthy subjects, which was absent in patients with MacTel Type 2.

### Follow-up Examinations

To assess disease-associated changes over time, follow-up examinations were performed in 28.6% of the eyes (n = 8). The average follow-up interval was 25 months, with a range from 6 months to 36 months. There was an average of 2.6 letter decrease in BCVA, which was statistically not significant.

**Fig. 6.**
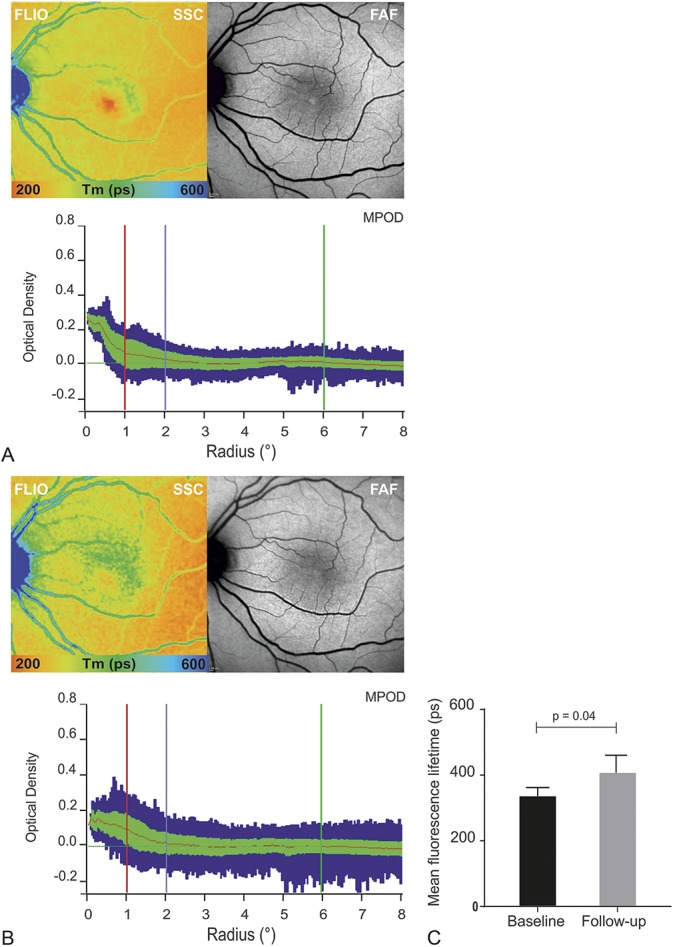
**A.** Disease progression within 24 months FLIO (SSC), fundus AF, and MPOD (baseline, left). Follow-up examination (right) shows clear disease progression with prolongation of temporal lifetimes and a reduction in the macular pigment density. **B.** Mean fluorescence lifetime of the short spectral of the temporal sector (T1) from the ETDRS grid. Mean and SD of Tm at baseline (left) and follow-up. **C.** Bar-graph showing progression of mean fluorescence lifetimes from the SSC from baseline to follow-up visit (r^2^ = 0.2480, *P* = 0.0409).

On follow-up, we observed a progression of prolonged fluorescence lifetimes, radiating from the center outward in an oval or round pattern (Figure 6A). Quantitative analysis of sector T1 showed a significant increase of Ƭm (22%) between initial and follow-up measurements (baseline SSC: 337 ± 13 ps vs. follow-up SSC: 411 ± 29 ps; r^2^ = 0.2480, *P* = 0.0409; Figure 6B).

## Discussion

Fluorescence lifetime imaging ophthalmoscopy is a relatively new imaging modality allowing to detect subtle changes within the retina and therefore provides additional information over other retinal imaging techniques.^14,15,18,19,22^ Advancements in retinal imaging over the past decade have increased our understanding of pathological features in MacTel. However, as clinical changes can be subtle, particularly in early stages of disease, imaging techniques to identify changes earlier and to monitor longitudinal changes noninvasively are important.

In our study of 28 eyes of patients with MacTel Type 2, unique features of mean retinal fluorescence lifetimes were identified, which are in keeping with a recently published report.^20^ We observed significantly prolonged lifetimes within the affected MacTel area, with the inferotemporal region showing the most pronounced changes with a general prolongation even in the very early stages of this disease. In addition, MPOD measurements correlated well with FLIO measurements, with both methods showing a loss of macular pigment with progression of the disease. The macular pigment typically encompasses the fovea, where a central well-circumcised round region of shortened lifetimes has been described as a normal finding in topographic FLIO maps.^18,19^ Despite the large variation of macular pigment concentration in the normal population, the pattern of distribution in MPOD measurements as well as in FLIO measurements is relatively uniform. A peak concentration is generally found at the foveal center with an eccentric decrease and fading at 8° and more. In patients with MacTel Type 2, we observed at later stages of the disease that the macular pigment lifetime signature was completely absent, and a marked oval-shaped irregular area of shortened lifetimes in a region of up to 4° to 7° surrounding the foveolar was identified. This was further illustrated using the phasor plot analysis demonstrating the central depletion of macular pigment (see **Figure 1, Supplemental Digital Content 1**, http://links.lww.com/IAE/A944). These findings are consistent with previous observations where retinal abnormalities have been suggested to present temporally to the fovea initially on fundus examination,^23^ imaging,^24^ and functional assessment using microperimetry.^25^ However, histopathological and clinicopathological studies of this distinct MacTel region confirmed findings of depleted macular pigment and Müller cell dysfunction.^6,7^

In contrast to FAF, in which lipofuscin is the predominant fluorophore in terms of fluorescence intensity in the retina, the FLIO technique has the ability to detect and characterize fluorescence lifetime data from other fluorophores, and their interaction with each other as well as the embedding matrix.^26^ However, there is still some controversy around the origin of the measured signal, and further clarification on the involved fluorophores is needed. To date, besides lipofuscin, collagen melanin, elastin, FAF, and NAD(P)H have been suggested to contribute to the fluorescence lifetime signal.^18^

An important finding of this study was a prolonged foveal and perifoveal lifetime pattern identified in MacTel eyes, which exceeded the prolongation that would be expected by macular pigment alone. Therefore, other factors seem to play a role in the observed fluorescence lifetime changes. Clinical characteristics such as intraretinal crystalline deposit accumulation, where we observed prolonged lifetimes outside areas of the macular pigment region, as well as hyperplastic retinal pigment epithelium migration and abnormalities in the juxtafoveolar retinal vessels may additionally contribute to the identified prolonged lifetimes. We have previously shown that ellipsoid zone loss with a preserved retinal pigment epithelium results in Ƭ_m_ prolongation.^15,18^ This association was also found in patients with MacTel (Figure 3). As intact photoreceptors have been shown to contribute to short lifetime signals, possibly due to generation of components of the visual cycle such as all-trans retinal, loss of photoreceptors will lead to longer lifetimes in MacTel patients.

Changes seen on FLIO at different disease stages seem disease specific for MacTel Type 2 and, as such, our study is supportive of the clinical applicability of this device in the diagnosis and monitoring of MacTel. In our study, longer lifetimes were significantly associated with more advanced clinical disease stages. Although in the early stages of MacTel we found subtle wedge-shaped prolongation of fluorescence lifetimes changes starting temporal to the fovea, with progressing disease, these changes involved the foveal center. Furthermore, fluorescence lifetime patterns change over time with enlargement of areas of long Ƭ_m_. Therefore, retinal autofluorescence lifetimes can be used to monitor disease progression and may serve as an objective readout to assess future therapeutic interventions.

This study has several limitations, including the patient number and heterogeneity between patients. This cohort included a larger number of patients with MacTel Type 2 in advanced stages with evidence of neovascularization. Because MacTel Type 2 is a relatively rare condition, more definite conclusions could be drawn with larger samples through multicenter studies.

## Conclusion

The characteristic lifetime changes in patients with MacTel Type 2, particularly in the inferotemporal region and the nasal–temporal difference, may be used as an additional marker for the clinical diagnosis and progression of the disease. The topographic FLIO maps offer a novel diagnostic tool in the investigation of eyes with MacTel and may provide information about macular pigment and possibly photoreceptor loss. Additional longitudinal data will be needed to determine the value of FLIO as a noninvasive screening, diagnostic imaging modality, and as a measure of disease progression in MacTel Type 2.

## Supplementary Material

SUPPLEMENTARY MATERIAL
