# Supplementary figures and images for: FLUORESCENCE LIFETIME PATTERNS IN MACULAR TELANGIECTASIA TYPE 2
Source: Retina. 2019 Jan 3;40(1):99–108. doi: 10.1097/IAE.0000000000002411 (PMC6924947; doi:10.1097/IAE.0000000000002411)

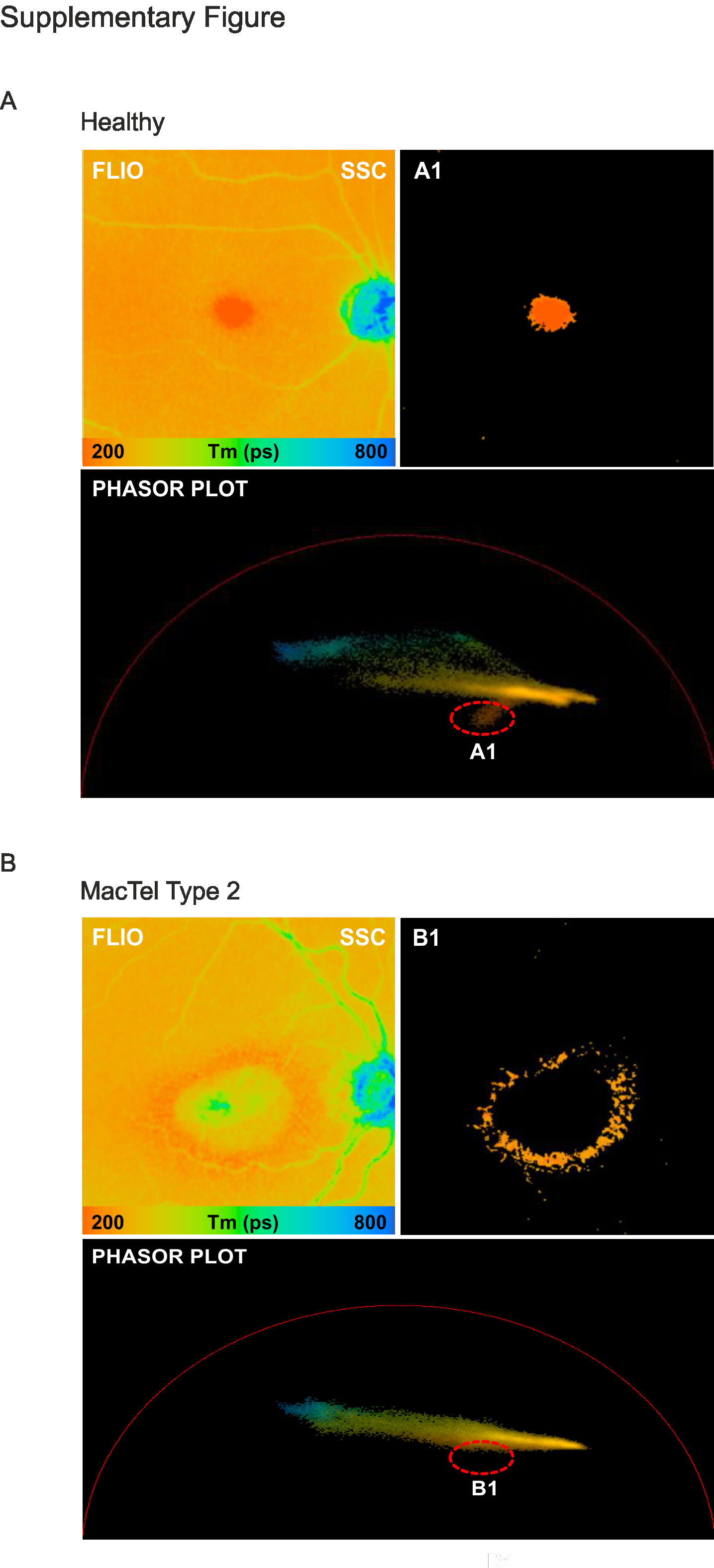

Supplement: SUPPLEMENTARY MATERIAL [file retina-40-99-s001.tif]
